# Hydrophobin-Based Surface Engineering for Sensitive and Robust Quantification of Yeast Pheromones

**DOI:** 10.3390/s16050602

**Published:** 2016-04-27

**Authors:** Stefan Hennig, Gerhard Rödel, Kai Ostermann

**Affiliations:** Institute of Genetics, Technische Universität Dresden, 01062 Dresden, Germany; Gerhard.Roedel@tu-dresden.de (G.R.); Kai.Ostermann@tu-dresden.de (K.O.)

**Keywords:** analyte detection, yeast pheromone, hydrophobin, surface functionalization, whole-cell biosensor, biosensor

## Abstract

Detection and quantification of small peptides, such as yeast pheromones, are often challenging. We developed a highly sensitive and robust affinity-assay for the quantification of the α-factor pheromone of *Saccharomyces cerevisiae* based on recombinant hydrophobins. These small, amphipathic proteins self-assemble into highly stable monolayers at hydrophilic-hydrophobic interfaces. Upon functionalization of solid supports with a combination of hydrophobins either lacking or exposing the α-factor, pheromone-specific antibodies were bound to the surface. Increasing concentrations of the pheromone competitively detached the antibodies, thus allowing for quantification of the pheromone. By adjusting the percentage of pheromone-exposing hydrophobins, the sensitivity of the assay could be precisely predefined. The assay proved to be highly robust against changes in sample matrix composition. Due to the high stability of hydrophobin layers, the functionalized surfaces could be repeatedly used without affecting the sensitivity. Furthermore, by using an inverse setup, the sensitivity was increased by three orders of magnitude, yielding a novel kind of biosensor for the yeast pheromone with the lowest limit of detection reported so far. This assay was applied to study the pheromone secretion of diverse yeast strains including a whole-cell biosensor strain of *Schizosaccharomyces pombe* modulating α-factor secretion in response to an environmental signal.

## 1. Introduction

Hydrophobins are low molecular weight proteins secreted by filamentous fungi. Despite their overall low sequence similarity, hydrophobins share a common structure, including a conserved pattern of eight cysteine residues which form four intramolecular disulfide bonds stabilizing the core protein structure [1,2,3,4,5]. Due to their amphipathic structure hydrophobins self-assemble into robust monolayers at hydrophobic-hydrophilic interfaces. According to the cysteine spacing pattern and the hydropathy plots, hydrophobins are divided into two classes reflecting the features of the monolayers, *i.e.*, their stability and morphology. Class I hydrophobins form highly stable protein layers characterized by a mosaic pattern of rodlets, which dissociate only under very harsh conditions, whereas class II hydrophobins form less resistant monolayers with crystalline packing. Due to their unique physicochemical properties hydrophobins fulfill a number of functions in their native hosts, e.g., they facilitate spore dispersal by providing hydrophobic spore coatings or they allow fungal hyphae to breach the interface to form aerial structures by reducing the surface tension of aqueous media [5,6,7,8].

Due to their ability to self-assemble into amphipathic monolayers, hydrophobins possess great potential for a variety of applications [2,5,9]. Solid supports functionalized with hydrophobins are biocompatible [10,11], showing very low friction at the nanoscale [12] and enhanced lubrication properties [13,14]. Hydrophobin-based surface engineering enables the immobilization of further proteins or protein domains (either via fusion proteins or via nonspecific protein adsorption to hydrophobin layers), e.g., the immobilization of enzymes and electroactive molecules at electrodes [15,16], *in vitro* patterning of proteins [17,18] or tailored functionalization of surfaces with enzymes, antibodies or DNA [19,20,21,22,23,24]. Moreover, the very high surface activity of hydrophobins offers the possibility to utilize them as emulsifiers or foam stabilizers in food industry, to solubilize and functionalize carbon nanotubes and nanoparticles or as a fusion tag for one-step protein purification upon heterologous expression in plants or fungi [2,5,9].

In this study, we employed a recombinant derivative of the class I hydrophobin EAS (also termed Bli-7 or Ccg-2) from *Neurospora crassa* [25,26]. We utilized hydrophobin-based surface engineering to quantify a small peptide, the *Saccharomyces cerevisiae* (*S. cerevisiae*) α-factor pheromone.

In its haploid state, cells of *S. cerevisiae* exist in one of two opposite mating types (termed a and α), which utilize peptide pheromones for communication. While α-type cells secrete a small unmodified peptide pheromone (α-factor), a-type cells release a small, post-translationally modified peptide (a-factor) to the environment [27]. Both cells types exhibit specific surface-exposed receptors for the pheromone secreted by cells of the opposite mating type, allowing for pheromone-based cell-cell communication as a part of the yeast′s sexual life cycle.

Yeast pheromones were utilized in previous experimental approaches to achieve synthetic cell-cell communication [28] or in biosensor designs to implement signal amplification [29]. In the approach described here we intended to employ the α-factor as a read-out signal for a novel type of whole-cell based biosensors. Whole-cell biosensors represent an emerging branch of biosensors utilizing living cells as the sensing device that respond to a certain analyte or environmental cue by modulating the expression of a reporter gene. Due to the ease of cultivation, manipulation and storage as well as their robustness, yeast cells are highly attractive for whole-cell biosensor applications [30,31]. Previously established yeast-based whole-cell biosensors primarily relied on the expression of fluorescent proteins, β-galactosidase or luciferase as reporter genes [30]. These proteins accumulate within the sensor cells, complicating optical signal read-out due to light scattering resulting from high sensor cell densities. In contrast, the α-factor is efficiently secreted into the cellular environment, hence the pheromone detection assay established in this study does not rely on intracellular protein accumulation. Using the pheromone as a read-out signal offers the perspective for signal transport, e.g., in microfluidic devices. Spatial separation of the sensor cells and the transducer element can be advantageous if the former are genetically engineered and have to be kept in safe compartments that only allow small molecules like the pheromone to exit. Additionally, intrinsic signal amplification can be achieved, as the pheromone is synthesized as a part of a large precursor protein which gives rise to four identical pheromone molecules upon maturation [32].

In this study, we utilized two derivatives of the recombinant EAS hydrophobin with one exposing the α-factor upon self-assembly at a hydrophobic surface, to immobilize a pheromone-specific antibody at the functionalized surface. Competitive detachment of the antibodies by applying soluble pheromone to the functionalized surface allows for quantification of the pheromone. The sensitivity of the assay could be tuned by adjusting the amount of pheromone-exposing hydrophobins within the protein layer. Furthermore, by using an inverse setup, we established a highly sensitive pheromone quantification device with lowest detection limit reported so far. This assay was exploited to study pheromone secretion of native and engineered yeast strains, including a sensor strain of *Schizosaccharomyces pombe* (*S. pombe*) that responds to extracellular thiamin (vitamin B1) concentrations. As thiamine is an important cofactor of several enzymes, and thiamine deficiency has been linked to several human health issues (including Alzheimer’s disease [33] and systolic heart failure [34]), measuring thiamine levels is of high importance in clinical and industrial scales. By taking use of a thiamine-responsive promotor to control pheromone synthesis, we created a novel whole-cell biosensor that utilizes the secreted peptide pheromone to generate a read-out signal.

## 2. Materials and Methods

### 2.1. Molecular Cloning of the Hydrophobin Genes EAS and EAS-α

*Escherichia coli* (*E. coli*) TOP10 F′ (Invitrogen, Darmstadt, Germany) was used for molecular cloning purposes. All constructs were obtained using standard molecular techniques [35]. The DNA sequence encoding the EAS hydrophobin without its N-terminal secretion signal was amplified and integrated into pET28b vector (Novagen, Darmstadt, Germany) 3′ of the sequence encoding the (His)_6_-tag, thus generating pET28b-EAS. To obtain a fusion protein of EAS and the yeast α-factor, the DNA sequences encoding a (GGGGS)_3_ linker element and the α-factor peptide were codon-optimized for expression in *E. coli* [36] and inserted 3′ of the EAS sequence in pET28b-EAS, resulting in pET28b-EAS-α.

### 2.2. Expression and Purification of Hydrophobins

pET28b-EAS and pET28b-EAS-α, respectively, were transformed into *E. coli* SHuffle^®^ T7 Express *lysY* (New England Biolabs, Frankfurt, Germany). Expression and purification of the hydrophobins were carried out essentially as described previously [37,38,39]. Briefly, transformants were grown in LB medium (1.0% (*w*/*v*) peptone, 0.5% (*w*/*v*) yeast extract, 0.5% (*w*/*v*) sodium chloride, pH 7.4) at 30 °C to the early log phase and expression of the hydrophobins was induced by addition of 0.4 mM isopropyl-β-d-thiogalactoside (IPTG). After 4 h, cells were harvested and washed twice using 50 mM Tris/HCl (pH 7.5). For hydrophobin purification, the cells were disrupted by lysozyme treatment (2 mg/mL) and sonication. Upon centrifugation (20,000× *g*, 4 °C, 10 min), recombinant hydrophobins were found in the pellet fraction and were solubilized in lysis buffer (50 mM sodium phosphate, 10 mM Tris, 8 M urea, pH 8.0). The proteins were purified by Ni^2+^ affinity chromatography (Novagen, Darmstadt, Germany) according to the manufacturer′s instructions. Finally, the hydrophobins were concentrated by ultrafiltration using a Vivaspin 20 column (5000 MWCO, Sartorius AG, Göttingen, Germany) and dialyzed twice for > 24 h against dialysis buffer (10 mM glutathione reduced, 1 mM glutathione oxidized, pH 5.4) according to Kwan *et al.* [38]. Final protein concentrations were determined via a Bradford assay [40]. Three μg of each purified protein fraction were separated by Tricine sodium dodecyl sulfate (SDS) polyacrylamide gel electrophoresis (PAGE) according to Schägger [41] and visualized by colloidal Coomassie staining or transferred to polyvinylidene fluoride (PVDF) membranes and probed with the (His)_6_-tag antibody (Roche Diagnostics, Mannheim, Germany) or the α-factor antibody (Peninsula Laboratories, San Carlos, CA, USA).

### 2.3. Surface Functionalization

To functionalize hydrophobic polystyrene surfaces, purified hydrophobins EAS and EAS-α were diluted in dialysis buffer to a final concentration of 2 μM and applied to the surfaces. Hydrophobins were allowed to self-assemble on the surface for 10 min at 80 °C [11,42], and excess solvent was carefully withdrawn. Functionalized surfaces were washed twice with water and twice with phosphate-buffered saline containing 0.01% (*v*/*v*) Tween-20 (PBST). Water contact angles were determined using a DSA10 Drop Shape Analysis System (Kruess, Hamburg, Germany) and the accompanying software (DSA 1.51.0.26). Additionally, some of the functionalized surfaces were treated with 2% (*w*/*v*) SDS at 80 °C for 10 min prior to contact angle measurement to analyze the hydrophobin layer stability.

### 2.4. Optimization of Hydrophobin Layer Composition

The hydrophobins EAS and EAS-α, respectively, were diluted in dialysis buffer to a final concentration of 2 μM and mixed in several molar ratios. 100 μL of the resulting hydrophobin solutions were used to functionalize individual wells of 96 well plates (unmodified polystyrene, Greiner Bio-One, Frickenhausen, Germany) according to the procedure described above. Residual protein binding sites of the wells were blocked with 1% (*w*/*v*) bovine serum albumin (BSA) in PBST for 1 h at ambient temperature (approximately 21 °C), and the functionalized surfaces were subsequently treated with 100 μL α-factor antibody solution (0.4 μg/mL in 0.5% (*w*/*v*) BSA in PBST) for 1 h at room temperature. Surfaces were washed four times using PBST prior to the application of 100 μL secondary antibody solution (GE Healthcare, Munich, Germany; 1:10,000 in 0.5% (*w*/*v*) BSA in PBST) coupled to horseradish peroxidase (HRP). After incubation for 1 h at ambient temperature, surfaces were washed four times using PBST and twice with 0.1 M sodium acetate (pH 5.2). Finally, the activity of the HRP was quantified by applying 100 μL of a substrate solution (0.1 mg/mL 3,3′,5,5′-tetramethylbenzidine, 0.01% (*v*/*v*) hydrogen peroxide, 0.1 M sodium acetate pH 5.2) and further incubation for 15 min prior to adding 100 μL of 2 M sulfuric acid. Absorbance at 450 nm was measured using an Infinite 200 instrument (Tecan, Männedorf, Switzerland). Data were normalized to absorbance values obtained for surfaces consisting of 100% EAS-α. All plotted values represent triplicate measurements of at least two independent experiments. Error bars indicate standard deviation.

### 2.5. Competitive Enzyme-Linked Immunosorbent Assay (ELISA)

To measure pheromone concentrations, an additional reaction step was included. After surface functionalization, blocking and treatment of the surfaces with the α-factor antibody (see above), the surfaces were washed four times using PBST prior to adding 100 μL of various dilutions of a synthetic α-factor (Zymo Research, Irvine, CA, USA; diluted in 0.5% (*w*/*v*) BSA in PBST) to individual wells. The plates were incubated for 1 h at 40 °C in a water bath to allow for the competition between the surface-immobilized α-factor (via fusion to the hydrophobin) and the dissolved α-factor to take place. Dissolved pheromone and detached antibodies were removed by washing the surfaces four times using PBST prior to subsequent application of 100 μL of the secondary antibody and the substrate solution (see above). Data were normalized to absorbance values obtained for non-pheromone treated surfaces. The limit of detection was calculated based on the mean values and three times the standard deviation of the antibody coverage obtained for control surfaces not treated with the pheromone.

Reusability of functionalized surfaces was analyzed by removing the antibodies after measuring the absorbance values. Functionalized surfaces (1.6% EAS-α) were used for competitive ELISA measurements, washed three times using PBST and antibodies were denatured by treatment with 300 μL stripping buffer (50 mM Tris/HCl, 1% (*w*/*v*) SDS, 50 mM 2-mercaptoethanol, pH 7.0) for 15 min at 55 °C. Subsequently, surfaces were washed four times using PBST, blocked with 1% (*w*/*v*) BSA in PBST at 4 °C overnight and used again for competitive ELISA measurements. All plotted values correspond to triplicate measurements of at least two independent experiments. Error bars indicate standard deviation.

### 2.6. Inverse ELISA

The α-factor antibody (0.4 μg/mL final concentration) was added to samples containing various concentrations of the synthetic α-factor (in 0.5% (*w*/*v*) BSA in PBST). The samples were incubated for 2 h at ambient temperature under constant rotation (30 rpm). Individual wells of 96 well plates were functionalized with hydrophobins and blocked with BSA (see above). 100 μL of the preincubated samples containing the pheromone and the antibody were applied to the functionalized surfaces and incubated for 2 h at 4 °C. The surfaces were washed four times using PBST prior to applying 100 μL of the secondary antibody and the substrate thereafter (see above). Data were normalized to absorbance values obtained for pheromone-free samples. Plotted values represent triplicate measurements of at least two independent experiments. Error bars indicate standard deviation.

### 2.7. Yeast Strains, Cultivation and Transformation

*S. cerevisiae* strains BY4741 and BY4742 (EUROSCARF, Frankfurt, Germany) as well as *S. pombe* strain HE620 (*h^+S^ leu1-32 ura4-D18*) [43] were used in this study. *S. cerevisiae* strains were grown in SD minimal medium (1.9 g/L yeast nitrogen base, 5 g/L ammonium sulfate, 20 g/L glucose). Edinburgh minimal medium (EMM) [44] was used for cultivation of *S. pombe*. Yeast transformations were carried out according to Gietz and Woods [45].

### 2.8. Measurement of α-Factor Concentrations in Yeast Culture Supernatants

Yeast cultures were inoculated in minimal medium at an initial cell density of approximately 5 × 10^6^ cells/mL. Samples were taken at the indicated time points, cells were removed by centrifugation (5000× *g*, 4 °C, 5 min), and 90 μL of supernatant were taken. Ethylene glycol tetraacetic acid (EGTA) and Tris/HCl buffer (pH 8.0) were added to the supernatant to a final concentration of 10 mM and 200 mM, respectively. Finally, Protease Inhibitor Cocktail (Roche Diagnostics, Mannheim, Germany) was added and samples were stored at −20 °C. The resulting samples (100 μL) were used to carry out inverse ELISA measurements as described above. All plotted values correspond to triplicate measurements of at least two independent experiments. Error bars indicate standard deviation.

## 3. Results and Discussion

### 3.1. Hydrophobin Design and Purification

**Figure 1:**
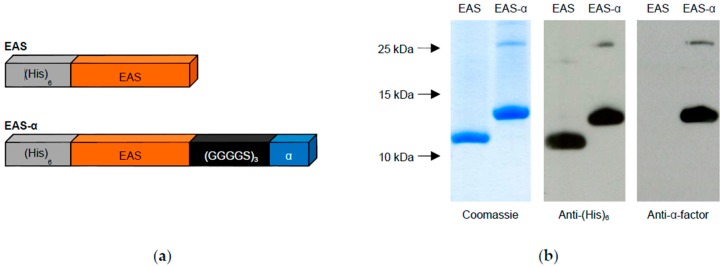
Hydrophobin design and purification. (**a**) Schematic illustration of the hydrophobins EAS and EAS-α. The EAS domain (orange) of the mature EAS hydrophobin from *Neurospora crassa* was fused to the (His)_6_-tag (gray) at its N-terminus for protein purification. In addition, EAS-α includes the sequence of the yeast α-factor (blue) fused to the C-terminus of the EAS domain via a flexible (GGGGS)_3_ linker (black); (**b**) Protein purification by Ni^2+^ affinity chromatography. Recombinant hydrophobins were purified as described in Materials and Methods, a sample was separated by Tricine-SDS-PAGE and visualized by colloidal Coomassie staining or immunologically detected by the indicated antibodies. Molecular weight is indicated at the left.

We intended to exploit hydrophobin-based surface engineering for the detection of the yeast α-factor pheromone as an example for the quantification of small proteins. To obtain functionalized surfaces exposing the α-factor, we employed a recombinant derivative of the class I hydrophobin EAS from *Neurospora crassa*. Two variants were generated (Figure 1a): the sequence encoding mature EAS hydrophobin, lacking the N-terminal signal peptide, was cloned into pET28b vector 3′ of the (His)_6_-tag coding region. A second construct, additionally carrying the sequences encoding a (GGGGS)_3_ linker and the α-factor peptide 3′ of the EAS sequence, was also integrated into pET28b vector. Both hydrophobins were expressed in the *E. coli* SHuffle^®^ T7 Express *lysY* strain. This strain was selected because it ensures the cytoplasmic formation of disulfide bonds, which play a crucial role in stabilizing soluble hydrophobins by preventing premature self-assembly in the absence of an interface [46,47]. Both hydrophobins were found in the pellet fraction of *E. coli* lysates upon centrifugation, but could be solubilized by 8 M urea and subsequently purified using the (His)_6_-tag [37,38,39]. A sample of the purified proteins was separated by Tricine-SDS-PAGE and probed with antibodies specific for the (His)_6_-tag or the α-factor peptide (Figure 1b) to ensure the integrity of the proteins. 

Coomassie staining revealed that the purified proteins exhibited the expected molecular weights. In the immunodetection, only EAS-α (harboring the pheromone tag) is recognized by the α-factor antibody, while both hydrophobins were detected with the (His)_6_-antibody due to their (His)_6_-tag. Except for a faint signal, most likely representing hydrophobin dimers, additional protein bands were barely visible, indicating a high degree of purity and stability (Figure 1b). Dimer formation has previously been reported for several class I hydrophobins, even upon separation in SDS-PAGE [48,49,50,51].

### 3.2. Surface Functionalization with Recombinant Hydrophobins

**Figure 2:**
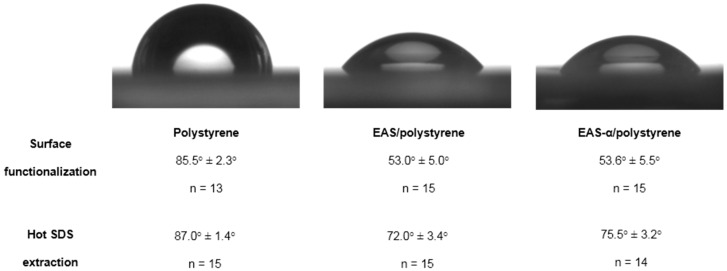
Water contact angle measurements of pure and hydrophobin-functionalized polystyrene surfaces. Polystyrene supports were functionalized with EAS and EAS-α as detailed in Materials and Methods and water contact angles were determined (images and **upper** panel). To analyze hydrophobin layer stability, the surfaces were additionally treated with hot SDS solutions prior to contact angle measurement (**lower** panel).

Upon self-assembly, hydrophobins are known to reverse the wettability of the substrate surface [2,5,9,37,52]. As reported in various studies, modifications both at the N-terminus and the C-terminus, as well as within the flexible loop structures of hydrophobins do not affect their self-assembling properties [10,11,53,54]. Even fusion of the highly polar (His)_6_-tag apparently has no adverse effect on hydrophobin self-assembly [37,42,55,56,57,58]. Consistent with these data, we could show by water contact angle measurements that the engineered hydrophobins retained the ability to self-assemble and to reverse the wettability of hydrophobic polystyrene (Figure 2).

Untreated polystyrene proved to be hydrophobic, showing contact angles of 85.5 ± 2.3°, whereas modification of polystyrene with EAS and EAS-α clearly decreased hydrophobicity, resulting in contact angles of 53.0 ± 5.0° and 53.6 ± 5.5°, respectively, thus demonstrating hydrophobin-based functionalization of the polystyrene surface (Figure 2). These values are similar to data reported for EASΔ15, a modified EAS variant lacking 15 amino acids in a flexible loop region, upon self-assembly at a hydrophobic substrate (56.0 ± 10.9° [39]). In contrast, functionalization with BSA caused only a minor decrease in the contact angle of polystyrene (76.1 ± 4.0°), indicating poor substrate adhesion. Upon treatment of hydrophobin-modified surfaces with hot SDS solutions, the contact angle increased slightly, indicating that some of the hydrophobins were extracted as reported for various class I hydrophobins previously [11,59,60,61,62].

In line with recent data, according to which 1.3 μM of the class II hydrophobin HFBI fused with dual chain avidin were able to saturate surface binding sites [21], we found that 2 μM of both EAS and EAS-α were sufficient to fully coat polystyrene surfaces (data not shown). Interestingly, a mixture of both hydrophobins shows similar self-assembling properties, indicating that EAS and EAS-α co-assemble. The formation of mixed layers of a hydrophobin carrying a fusion partner and the respective unmodified variant as a molecular spacer has been reported previously [19,22,63]. Moreover, class I and class II hydrophobins have been found to co-assemble at the same surface [64].

### 3.3. Optimization of Hydrophobin Layer Composition

**Figure 3:**
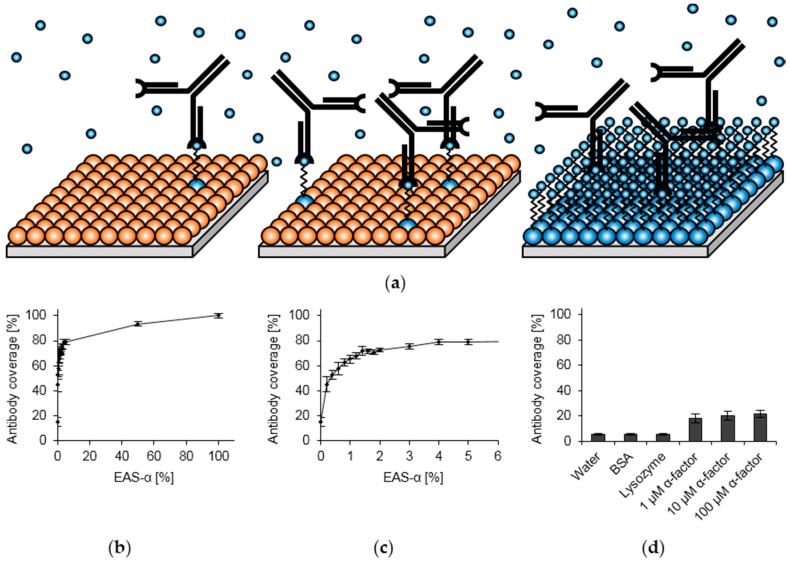
Optimization of hydrophobin layer composition. (**a**) Schematic drawing of monolayers consisting of EAS (**orange**) and EAS-α (**blue**) in different ratios to illustrate the concept of antibody coverage and sensitivity. Antibodies and pheromones are depicted in as black Y-shaped structures and small blue spheres, respectively; (**b**) Effect of EAS-α amount on antibody coverage. Hydrophobic polystyrene surfaces were functionalized with EAS and EAS-α in different molar ratios and subsequently treated with the α-factor antibody and a secondary antibody as detailed in Materials and Methods; (**c**) Enlarged image section of (**b**) to visualize the effect of low EAS-α amount on antibody coverage; (**d**) Antibody coverage obtained for surfaces treated with water, control proteins or the bare α-factor instead of hydrophobins.

To optimize the hydrophobin layers for the desired biosensing application, two parameters were considered. A high number of antibodies immobilized per surface area (“high antibody coverage”) is desirable to maximize the signal. This requires a high number of surface-exposed binding sites, although steric hindrance between the antibodies may prevent that all binding sites can be occupied. On the other hand, as our assay relies on competitive detachment of antibodies from the surface, an excess of antibody binding sites at the surface might be detrimental to the assay sensitivity. Higher pheromone concentrations are required to remove the antibodies from the surface. Optimal surface functionalization would thus allow for maximal antibody coverage with a minimum of surface-exposed binding sites (Figure 3a).

To achieve this goal, we used different molar ratios of EAS and EAS-α to functionalize polystyrene surfaces and applied the α-factor antibody to the surfaces. The antibody coverage was determined by use of a secondary antibody coupled to HRP, whose activity can easily be quantified colorimetrically. For high amounts of EAS-α, an effect on the antibody coverage was barely visible, as can be seen by the almost horizontal curve in a wide range of EAS-α concentrations (Figure 3b). This observation may indicate that, due to the large difference in the size of IgG antibodies (approximately 14 nm × 10 nm × 5 nm [65]) and hydrophobin monomers (2.7 nm in diameter [38]), not all of the surface-exposed binding sites can be occupied. Attachment of the antibodies might be limited by steric effects between antibodies. Only in a narrow concentration range, the antibody coverage increases with higher EAS-α concentration (up to 2%, Figure 3c). For a molar EAS-α content up to 1.6%, at which 71.7% ± 1.9% of the maximum antibody coverage were reached, a rather steep increase in antibody coverage with increasing EAS-α concentration was observed. In contrast, the steepness was clearly reduced upon further increasing the EAS-α content. We therefore consider 1.6% as the optimal concentration of EAS-α for the biosensing application. When surfaces were coated exclusively with synthetic α-factor or control proteins, antibody coverage was low (Figure 3d), underscoring the benefit of hydrophobin-based surface engineering. Notably, even surfaces functionalized with 100% EAS without the pheromone tag resulted in remarkably high antibody coverage values (15.0% ± 3.6%), probably reflecting electrostatic attractive forces between the antibody and the hydrophobin layer as previously observed [23,24,66].

### 3.4. Competitive ELISA

**Figure 4:**
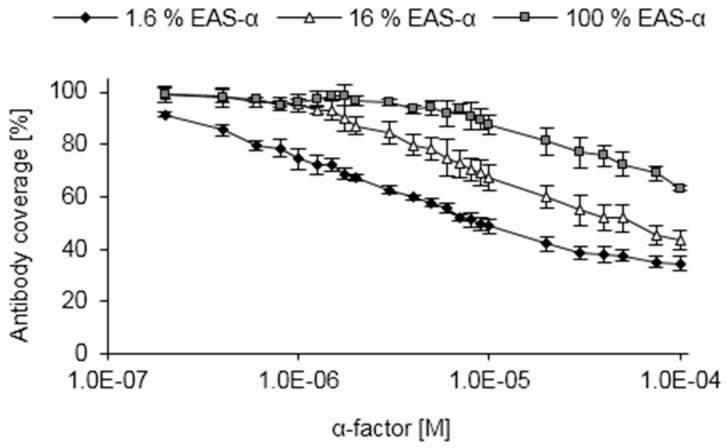
Calibration curves of the competitive ELISA. Polystyrene surfaces were functionalized with EAS and EAS-α in different molar ratios and used for competitive ELISA calibration by applying various concentrations of synthetic α-factor. Competition was performed at 40 °C to enhance sensitivity.

In order to apply hydrophobin-based surface engineering for quantification of the α-factor pheromone, polystyrene surfaces were functionalized as described above with a mixture of EAS and EAS-α, followed by application of α-factor antibodies to occupy surface-exposed binding sites. Subsequently, synthetic pheromone was added to allow for competition with the α-factor molecules immobilized via EAS-α, resulting in the detachment of antibodies. The remaining antibodies were quantified by use of a secondary antibody (Figure 4).

In line with the competitive removal of the antibodies from the surface, increasing pheromone concentrations caused a gradual decrease in antibody coverage (Figure 4). When the optimized surface composition of 1.6% EAS-α was employed, a lower limit of detection in the range of 0.2 μM α-factor was obtained. Functionalized surfaces with a higher amount of EAS-α showed an altered sensitivity. Far higher pheromone concentrations (about 2 μM and 10 μM for surfaces consisting of 16% EAS-α and 100% EAS-α, respectively) were required to obtain a significant decrease in antibody coverage for these surfaces. These data show that the sensitivity of the assay can be predefined by adjusting the percentage of pheromone-exposing hydrophobins used to functionalize the surface. However, due to the low absorbance values obtained, further lowering the EAS-α concentration below 1.6% EAS-α does not result in an elevated sensitivity of the assay (data not shown). Remarkably, even very high pheromone concentrations were insufficient to completely remove the antibodies from the functionalized surfaces, most likely reflecting non-specific interactions between the antibodies and the hydrophobin layer (see above). While this might reduce the sensitivity in the case of very high pheromone concentrations, it can be overcome by adjusting the sensitivity of the assay with increasing EAS-α content of the hydrophobin layer.

Hydrophobins have been used previously to immobilize antibodies on solid supports, taking use of non-specific interactions between the hydrophobin layer and the antibodies [23,24,66]. Asakawa *et al.* [19] employed a fusion protein of class II hydrophobin HFBII with the maltose binding protein (MBP), which was integrated into a monolayer using unmodified HFBII as a molecular spacer. By this approach, a MBP-specific antibody could be specifically immobilized at the functionalized surface to investigate layer homogeneity. The competitive detachment of the antibodies by addition of soluble MBP was not reported.

### 3.5. Performing the Competitive ELISA Under Varying Conditions

We next investigated the sensitivity of the assay towards chemical and physical parameters during the competition step (Figure S1). We tested the competition efficiency of the EAS/EAS-α-based assay under various parameters (pH, ionic strength, concentration of detergent). Surprisingly, none of these parameters affected the competition efficiency (Figure S1). Although the absorbance values were severely reduced in an acidic environment, the competition efficiency was not altered. The reduced binding efficiency of antibodies in acidic environments is probably caused by electrostatic repulsive forces between the antibodies (which are positively charged under these conditions) and the positively charged (His)_6_-tag exposed by the hydrophobin layer. This is in line with previous studies suggesting that antibodies could be immobilized most efficiently on hydrophobin layers under pH conditions ensuring that antibodies and hydrophobin layers are oppositely charged [23,24,66].

We next tested the possibility to enhance the assay sensitivity by physical parameters during the competition step. Indeed, when the competition was performed at elevated temperatures (up to 40 °C), a significant increase in the sensitivity was observed (Figure S1). The increased sensitivity at elevated temperatures might result from weakening the interactive forces between the antibody and its target, but it might also reflect an increase in diffusion. In support of the latter hypothesis, increasing the viscosity of the solvent by adding sucrose led to a marked reduction in sensitivity (Figure S1). We conclude that the diffusion of pheromones to the surface and diffusion of detached antibodies into the bulk solution (both being driven by Brownian motion) are of utmost importance to define the sensitivity, while the interactive forces between the antibody and the pheromone are of minor importance. Thus, the assay allows for robust sensing of the pheromone largely independent on the composition of the sample matrix.

### 3.6. Reusability of Functionalized Surfaces

**Figure 5:**
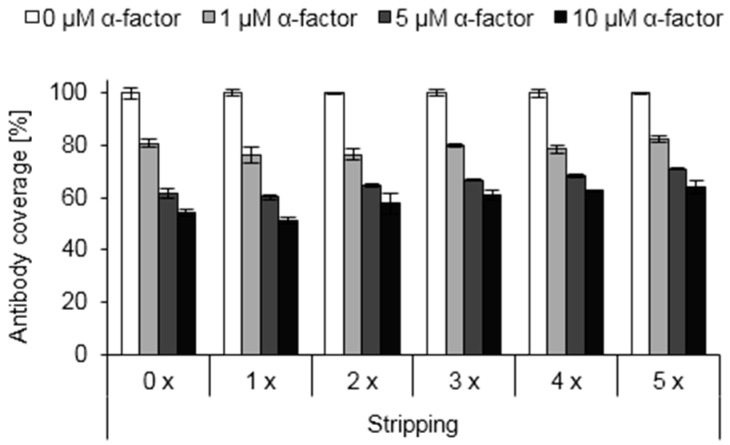
Reusability of functionalized surfaces. Polystyrene surfaces were functionalized with hydrophobins (1.6% EAS-α) and used for competitive ELISA measurements. After measuring the absorbance values, antibodies were stripped off (see Materials and Methods) and functionalized surfaces were utilized for further measurements the next day.

The highly robust class I hydrophobin monolayers can only be dissolved under very harsh conditions, e.g., by treatment with 100% trifluoroacetic acid [2,3,5]. We therefore tested whether the functionalized surfaces can be reused by selectively removing the bound antibodies. Indeed, we found that the antibodies can be denatured without harming the hydrophobin layers, thus providing the opportunity to use the functionalized surfaces multiple times (Figure 5).

While the sensitivity of the competitive ELISA was barely affected by multiple stripping events, a 30%–40% reduction in signal height was noticed after the first stripping cycle (Figure S2). No further signal reduction was observed upon further rounds of stripping. Contact angle measurements indicate that the initial signal reduction may result from a partial removal of EAS and EAS-α by the treatment with hot SDS (Figure 2), in line with previous observations for various class I hydrophobins [11,59,60,61,62]. However, our data show that the hydrophobin layers are sufficiently robust to allow for multiple measurements with similar sensitivity.

### 3.7. Inverse ELISA

**Figure 6:**
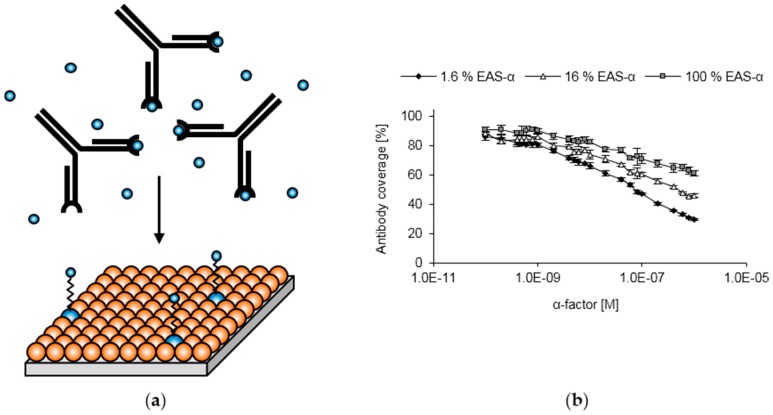
Concept and calibration of the inverse ELISA. (**a**) Schematic illustration of the inverse ELISA setup. In a first step, antibodies (black) are premixed with a sample containing the pheromone (small blue spheres) to occupy some of the binding sites of the antibodies. Subsequently, the antibody-pheromone mixture is applied to the functionalized surfaces. Only antibodies that still exhibit free binding sites are able to attach to the surface; (**b**) Calibration of the inverse ELISA. Several dilutions of the synthetic α-factor were premixed with the α-factor antibody. Afterwards, these samples were applied to polystyrene surfaces functionalized with EAS and EAS-α in different molar ratios.

The high affinity between antibodies and their antigens (dissociation constants in the nanomolar to picomolar range [67,68,69]) may limit the sensitivity of the competitive assay. We therefore investigated whether measurements with increased sensitivity can be enabled by an inverse assay (Figure 6a). In this setup, the antibodies are premixed with pheromone-containing samples. Subsequently, the samples are applied to functionalized surfaces, allowing for the attachment of antibodies that still carry free binding sites. Competition between surface-exposed pheromones and pheromones in bulk solution is largely suppressed by performing the second step at 4 °C. Bound antibodies are quantified by the use of the secondary antibody. A comparable approach was previously used to directly quantify hydrophobins [70].

As expected, increasing pheromone concentrations led to a gradual reduction in antibody coverage (Figure 6b). Compared to the competitive assay, the inverse assay is more sensitive, with a lower detection limit in the range of 0.1 nM pheromone. Again, the sensitivity of the assay could be predefined by the choice of the surface composition (*i.e.*, by adjusting the EAS-α amount). The inverse assay proved to be similarly robust against changes in pH, ionic strength or detergent concentration (Figure S3).

With a detection limit of 0.1 nM, the inverse assay is the most sensitive pheromone quantification method reported so far. So far, most approaches to quantify the pheromone were based on the pheromone-driven physiological activity, e.g., expression of fluorescent proteins, of specifically engineered reporter strains [71,72,73,74]. However, this approach requires at least 5–10 nM pheromone and reproducible conditions during the pheromone treatment. Recently, an alternative approach to quantify yeast pheromones was reported [75], in which the peptide pheromones were non-specifically adsorbed on a modified polystyrene surface and subsequently detected by a pheromone-specific antibody. While this assay allowed the quantification of pheromones in a concentration as low as 1.2 nM, the measurement requires significantly more time compared to the assays developed here. Moreover, repeated use of the surfaces was not reported. Other approaches based on mass spectrometry [76] or HPLC (data not shown) proved to be less sensitive.

### 3.8. Pheromone Secretion of S. cerevisiae Strains

Due to its high sensitivity and robustness, the inverse ELISA is ideally suited to measure pheromone concentrations in yeast culture supernatants. As yeast cells rapidly acidify their environment, and low pH values affect the maximum signal height (Figures S1 and S3) and eventually cause denaturation of the antibodies, we adjusted the supernatants to pH 8.0. Furthermore, protease inhibitors were added to prevent proteolytic degradation of the antibodies by proteases secreted by yeast cells or released upon cell lysis.

**Figure 7:**
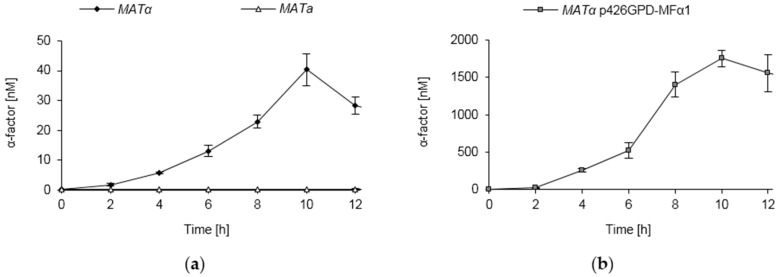
Pheromone secretion of wild type and engineered *S. cerevisiae* strains. (**a**) Pheromone secretion of wild type strains. The strains BY4741 (*MAT a*) and BY4742 (*MAT α*) were grown in minimal medium. At the indicated time points, samples of the culture supernatant were taken and pheromone concentrations were determined by inverse ELISA measurements; (**b**) Pheromone secretion of a *S. cerevisiae* strain engineered for high pheromone secretion. The strain BY4742 (*MAT α*) carrying the plasmid p426GPD-MFα1 was grown in minimal medium. Samples were taken at the indicated time points and analyzed by inverse ELISA measurements.

Pheromone concentrations in the supernatants of α-type cells were determined by sampling growing cultures over time. The wild type strain BY4742 was found to accumulate about 30–50 nM α-factor within 10 h of growth, thereafter the pheromone concentrations declined (Figure 7a). As α-type cells do not secrete the pheromone-degrading protease Bar1p [77], this might be caused by non-specific proteolysis of the pheromone. As expected, the supernatant of the a-type strain BY4741 did not contain any α-factor (Figure 7a).

Furthermore, yeast cells of the mating type α were engineered to secrete high amounts of the α-factor by overexpressing *MFα1* under the control of the strong *GPD* promoter. Wild type yeast cells harbor two α-factor coding genes (*MFα1* and *MFα2*), but 90% of the α-factor released can be ascribed to *MFα1* expression [75]. The pheromone overexpressing strain accumulated very high pheromone concentrations, with maximum levels of 1–2 μM within 10–12 h of incubation (Figure 7b). Occasional deviations in individual cultures might be attributed to the plasmid-based expression system and the resulting inhomogeneity in expression levels of individual cells [78]. Taken together, these data unambiguously show that the ELISA-based assay is able to specifically detect the pheromone even in complex sample matrices such as yeast culture supernatants.

### 3.9. Pheromone Secretion of Engineered S. pombe Cells

**Figure 8:**
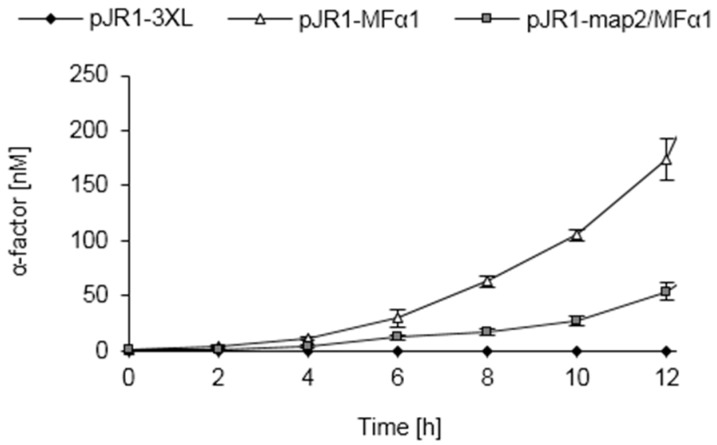
Pheromone secretion of engineered *S. pombe* strains. *S. pombe* HE620 was transformed with plasmids pJR1-3XL, pJR1-MFα1 or pJR1-map2/MFα1 [82] and grown in minimal medium. At the indicated time points, samples of the culture supernatants were taken and pheromone concentrations were measured by the inverse ELISA technique.

Fungal pheromones can be functionally expressed in non-authentic hosts to characterize fungal pheromone systems or to achieve synthetic inter-species communication [79,80,81,82,83,84]. In a previous study we reported the heterologous expression of *S. cerevisiae* α-factor in *S. pombe*. The activity of the secreted pheromone was determined by the response of engineered reporter cells [82]. Here we quantified the pheromone secreted by the engineered *S. pombe* strains (Figure 8). *S. pombe* cells carrying an empty vector (pJR1-3XL), that served as a negative control, were not found to secrete any α-factor, again highlighting the selectivity of the ELISA-based approach. Genetically engineered cells expressing the *S. cerevisiae MFα1* gene under the control of the strong *nmt1* promoter accumulated about 150–300 nM and 900–1500 nM α-factor, respectively, in the supernatants after 12 h and 24 h cultivation. In contrast to α-type cells of *S. cerevisiae* (see above), there was no evidence for proteolytic degradation of the pheromone. In the previous study [82], a high pheromone activity was observed with engineered *S. pombe* cells expressing a chimeric *map2*/*MFα1* gene. In this construct, two of the four P-factor repeats of the *S. pombe map2* gene (encoding the *S. pombe* P-factor pheromone precursor) were exchanged by the coding sequence of α-factor (Figure S4). Remarkably, we found that the respective *S. pombe* transformants accumulated about 30–50 nM and 400–600 nM within 12 h and 24 h, respectively (Figure 8). Most likely, the difference in pheromone secretion between strains expressing *MFα1* or *map2*/*MFα1* reflects the different numbers of α-factor copies (four copies in *MFα1 vs.* two copies in *map2*/*MFα1*; Figure S4).

The detection of higher pheromone levels in *S. pombe* cells expressing *MFα1* compared to cells expressing *map2*/*MFα1* is in marked contrast to the data of the previously reported pheromone activity of the respective supernatants, according to which the α-factor activity is significantly higher in the supernatants of *map2*/*MFα1* expressing strains [82]. We assume that this difference can be explained by incomplete N-terminal processing of the α-factor precursor in the *S. pombe* (Figure S5). In contrast, the chimeric *map2*/*MFα1* gene encodes the intrinsic processing sites of the *S. pombe* P-factor precursor and is hence expected to undergo efficient maturation, giving rise to fully processed and active α-factor.

### 3.10. Establishment of a Functional Whole-Cell Biosensor

**Figure 9:**
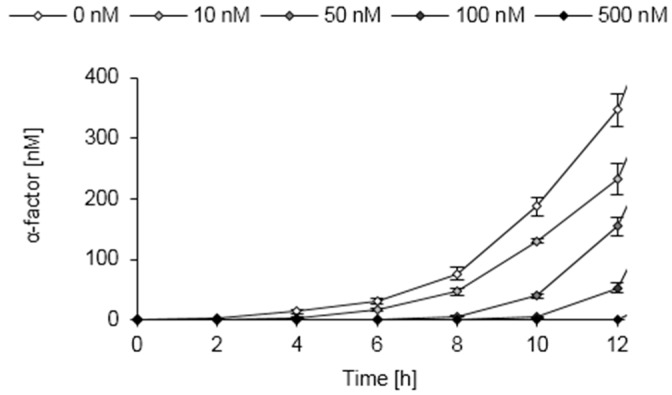
Pheromone secretion of a whole-cell biosensor strain of *S. pombe* responding to extracellular thiamine concentrations. *S. pombe* HE620 was transformed with pJR1-MFα1 and grown in minimal medium supplied with different amounts of thiamine (0–500 nM). Pheromone secretion was analyzed by inverse ELISA measurements of the culture supernatants sampled at the indicated time points.

We used the engineered *S. pombe* strains to establish a proof of principle whole-cell biosensor by utilizing the secreted peptide pheromone to create a read-out signal. To this end, the *nmt1* promoter of *S. pombe,* which can be tightly regulated by the thiamine (vitamin B1) concentration of the extracellular environment [85], was used. In the absence of thiamine, the *nmt1* promoter mediates high expression levels, whereas it shows only minor activity in the presence of thiamine. In a narrow range of thiamine concentrations, the activity of the *nmt1* promoter can be regulated gradually [86,87], which we used here to establish a whole-cell biosensor for thiamine. The engineered *S. pombe* strain expressing *MFα1* under the control of the *nmt1* promoter was exogenously supplied with different concentrations of thiamine, and the concentration of the secreted pheromone was analyzed (Figure 9).

With increasing thiamine concentration, the strain secreted gradually less pheromone, in line with the thiamine-dependent downregulation of the *nmt1* promoter. After 4 h incubation with thiamine, the pheromone secretion was decreased compared to untreated cells (Figure 9). This result confirms the data of Maundrell [85] that full repression of the *nmt1* promoter by thiamine requires about 3 h. The novel whole-cell biosensor is able to detect thiamine concentrations as low as 10 nM, which is comparable to previously established whole-cell and enzyme-based biosensors for thiamine [88,89]. Employing the yeast pheromone as a read-out signal, we thus developed a whole-cell biosensor that benefits from intrinsic signal amplification, as multiple pheromone copies are released from a single precursor molecule (Figure S4).

Furthermore, signal transport of the secreted and soluble α-factor might be feasible. Within a microfluidic device, the pheromone might be transported from the sensor cells to the transducer device in a fluid stream, allowing for spatial separation of the biological components and the transducer element.

## 4. Conclusions

In this study we report on a class I hydrophobin (EAS)-based surface functionalization that allows for highly sensitive quantification of small molecules as exemplified with the yeast pheromone α-factor. Furthermore, this approach was applied to implement a novel kind of whole-cell biosensor exploiting the secreted peptide pheromone as a read-out signal. By varying the ratio of EAS to EAS-α hydrophobins, the sensitivity of the pheromone quantification assay could be accurately adjusted, thus providing the possibility to control the dynamic range of pheromone concentrations to be measured. Furthermore, the highly robust class I hydrophobin layers allowed multiple use of the functionalized surfaces. The inverse ELISA proved to be the most sensitive technique for pheromone quantification that has been reported so far. It enables to quantify the pheromone secreted by different yeast strains and to set up a novel whole-cell biosensor based on engineered *S. pombe* cells that modulate pheromone secretion in response to an environmental cue.

Application of the hydrophobin-based biosensing platform is not restricted to quantification of the yeast pheromone, but offers a vast variety of (small) peptides to be quantified. Preliminary results suggest that haemagglutinin (HA)-tagged hydrophobins allow for quantifying HA-fusion proteins reliably (data not shown). Hydrophobins can be specifically modified, either genetically by the use of recombinant hydrophobins [19,21,22,37,63] or chemically by covalent modification with fluorophores [54,90] or dendritic macromolecules [20]. We envisage that this work may provide a more general platform to enable small molecule sensing. Hydrophobins can be engineered to expose user-defined small target structures at a functionalized surface, and respective antibodies or aptamers designed to specifically recognize these target molecules may be utilized to allow for the quantification of target-bearing molecules.

## Supplementary Materials

The following are available online at http://www.mdpi.com/1424-8220/16/5/602/s1, Figure S1: Performing the competitive ELISA under varying conditions, Figure S2: Effect of repeated antibody stripping on the signal height, Figure S3: Performing the inverse ELISA under varying conditions, Figure S4: Structure of authentic and chimeric pheromone genes, Figure S5: Selectivity of the ELISA-based pheromone quantification assay.

## Abbreviations

The following abbreviations are used in this manuscript:

*S. cerevisiae*	*Saccharomyces cerevisiae*
*S. pombe*	*Schizosaccharomyces pombe*
*E. coli*	*Escherichia coli*
IPTG	isopropyl-β-d-thiogalactoside
SDS	sodium dodecyl sulfate
PAGE	polyacrylamide gel electrophoresis
PVDF	polyvinylidene fluoride
PBST	phosphate-buffered saline containing 0.01% (*v*/*v*) Tween-20
BSA	bovine serum albumin
HRP	horseradish peroxidase
ELISA	enzyme-linked immunosorbent assay
EMM	Edinburgh Minimal Medium
EGTA	ethylene glycol tetra-acetic acid
MBP	maltose binding protein
HA	haemagglutinin
