# Supplementary material for: Hydrophobin-Based Surface Engineering for Sensitive and Robust Quantification of Yeast Pheromones
Source: Sensors (Basel). 2016 Apr 27;16(5):602. doi: 10.3390/s16050602 (PMC4883293; doi:10.3390/s16050602)
Supplement: Supplementary file 1 [file sensors-16-00602-s001.pdf]

Supplementary Material: Hydrophobin-Based Surface Engineering for Sensitive and Robust Quantification of Yeast Pheromones

Stefan Hennig, Gerhard Rödel and Kai Ostermann

S1. Performing the Competitive ELISA under Varying Conditions

We analyzed the sensitivity of the competitive ELISA towards chemical and physical parameters during the competition step by modifying the competitive ELISA protocol. Polystyrene surfaces were functionalized with hydrophobins (1.6% EAS-α), blocked and treated with α-factor antibody (see Materials and Methods). Subsequently, the surfaces were washed four times with PBST and twice with the indicated buffer prior to applying 0 µM or 10 µM of α-factor dissolved in 0.5% (*w*/*v*) BSA in the indicated buffer. After incubation for 1 h at room temperature (unless otherwise indicated), the surfaces were washed four times with the indicated buffer and twice with PBST before proceeding with secondary antibody treatment as in the competitive ELISA.

Surprisingly, the competition efficiency was not affected by changes in the pH, ionic strength or detergent concentration during the competition step. Although the absorbance values were clearly reduced in acidic conditions, we did not observe any effect on the competition efficiency (Figure S1a,b). The reduced binding efficiency of antibodies in acidic surroundings might be a consequence of electrostatic repulsive forces between the antibodies (which are positively charged in acidic conditions) and the positively charged $(His)_6$-tag exposed by the hydrophobin layer. This is consistent with previous studies suggesting that the immobilization of antibodies on hydrophobin layers is most efficient if antibodies and hydrophobin layers are oppositely charged [1–3]. Modifying the ionic strength or the detergent concentration during the competition step did not have any effect on the absorbance values or the competition efficiency (Figure S1c,d). We therefore conclude that the competitive ELISA assay is largely robust against changes in the sample matrix composition.

We also assessed whether the assay sensitivity could be enhanced by modifying the physical parameters during the competition step. Indeed, when the competition step was performed at elevated temperatures (up to 40 °C), a significant increase in the sensitivity towards the pheromone was observed (Figure S1e). Higher temperatures dramatically decreased the absorbance values, presumably due to antibody denaturation (data not shown). In contrast, upon increasing the viscosity of the solvent by adding sucrose, we observed a clear reduction in sensitivity (Figure S1f). We thus conclude that the diffusion of pheromones to the surface and diffusion of detached antibodies into the bulk solution (both processes are controlled by Brownian motion) are most important to define the sensitivity. In marked contrast, the interactive forces between the antibody and the pheromone are of minor importance to define the sensitivity. Thus, the assay allows for robust sensing of the pheromone highly independent on the sample matrix composition.

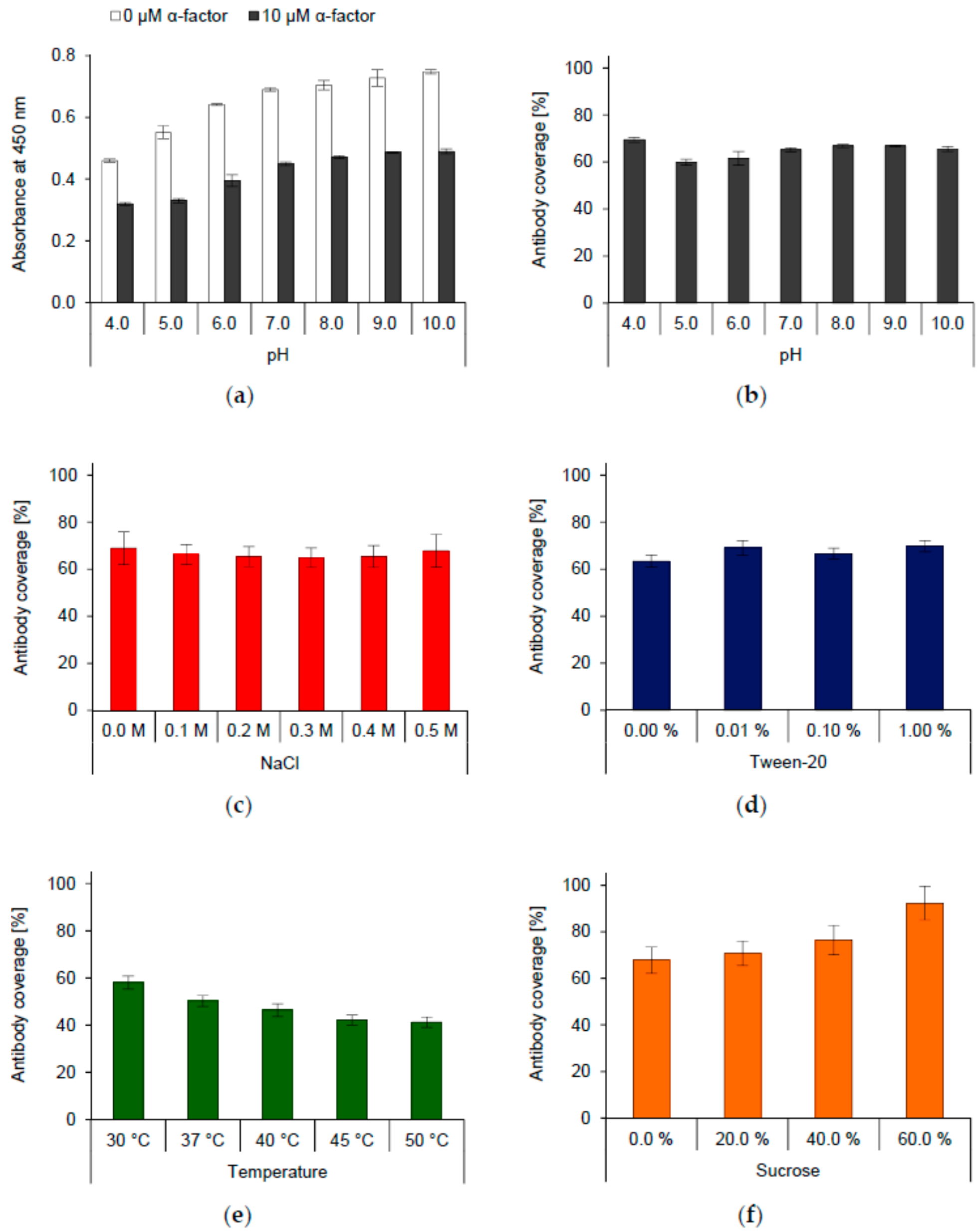

Figure S1. The competitive ELISA under varying conditions. (**a**) Influence of sample pH on the absorbance values determined. All buffers contained 0.1 M sodium chloride and 0.01% (*v*/*v*) Tween-20; (**b**) Normalized data regarding the influence of the sample pH. For data normalization, values corresponding to non-pheromone treated surfaces were set to 100% antibody coverage and values corresponding to surfaces treated with 10 µM of the pheromone are plotted; (**c**) Influence of the ionic strength on the competitive ELISA. Different amounts of sodium chloride were added to phosphate buffer (pH 7.0) containing 0.01% (*v*/*v*) Tween-20. Data were normalized according to (**b**); (**d**) Influence of detergent concentration on the competitive ELISA. Different amounts of Tween-20 were added to phosphate buffers (pH 7.0) containing 0.1 M sodium chloride. Data were normalized according to (**b**); (**e**) Influence of the temperature during the competition step on the competitive ELISA. Phosphate buffers (pH 7.0) containing 0.1 M sodium chloride and 0.01% (*v*/*v*) Tween-20 were used. The functionalized surfaces were stored in a water bath at the indicated temperature during the competition step. Data were normalized according to (**b**); (**f**) Influence of the sample viscosity on the competitive ELISA. Different amounts of sucrose were added to phosphate buffer (pH 7.0) containing 0.1 M sodium chloride and 0.01% (*v*/*v*) Tween-20. Data were normalized according to (**b**). All plotted values correspond to triplicate measurements of at least two independent experiments. Error bars indicate standard deviation.

S2. Effect of Repeated Antibody Stripping on the Signal Height

Class I hydrophobins form highly robust protein monolayers that require treatment with strong acids, e.g., 100% trifluoroacetic acid, to dissociate [4–6]. We therefore tested whether we can selectively remove the antibodies attached to a functionalized surface without harming the hydrophobin layer. Functionalized surfaces (1.6% EAS-α) were used for competitive ELISA measurements and antibodies were stripped off as detailed in Materials and methods. To analyze the effects of repeated stripping cycles on the competitive ELISA performance, we each time included freshly functionalized surfaces and treated these similar to the stripped ones. The sensitivity of the competitive ELISA assay was barely affected by repeated stripping events (Figure 5). In order to analyze whether the signal height is altered upon repeated stripping, we plotted the signal ratio (absorbance values of stripped surfaces compared to freshly prepared surfaces, both treated with similar pheromone concentrations, Figure S2).

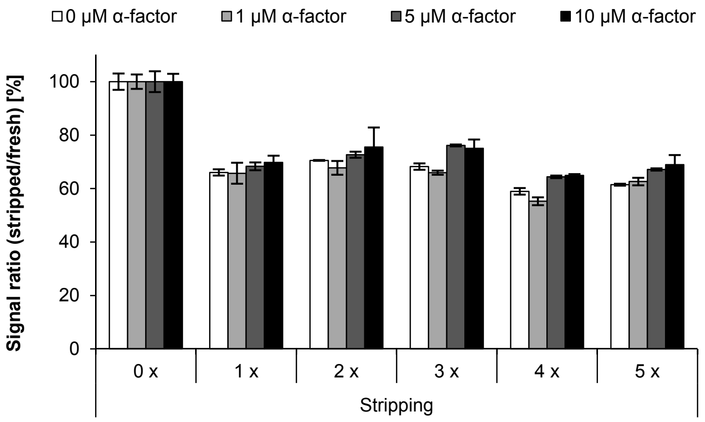

Figure S2. Effect of repeated antibody stripping on signal height. The signal ratio of stripped surfaces was plotted. A representative set of data, corresponding to triplicate measurements, is shown. Error bars indicate standard deviation.

About 30%–40% reduction in the signal height was evident after the first stripping event, whereas further stripping cycles did not affect the signal height further (Figure S2). The initial decrease in signal height might be attributed to partial extraction of the hydrophobins by the hot SDS treatment, a phenomenon frequently observed for class I hydrophobins [7–11]. However, further stripping cycles did not cause further hydrophobin extraction, thus highlighting the stability of class I hydrophobin assemblies.

S3. Performing the Inverse ELISA under Varying Conditions

The influence of varying chemical parameters on the inverse ELISA was analyzed (Figure S3). To this end, the buffers used for the initial binding step were modified. The α-factor antibody was incubated with 0 nM or 100 nM of the pheromone in 0.5% (*w*/*v*) BSA in the indicated buffer for 2 h at room temperature prior to applying the mixture to the functionalized surfaces and proceeding with the inverse ELISA as described in Materials and Methods.

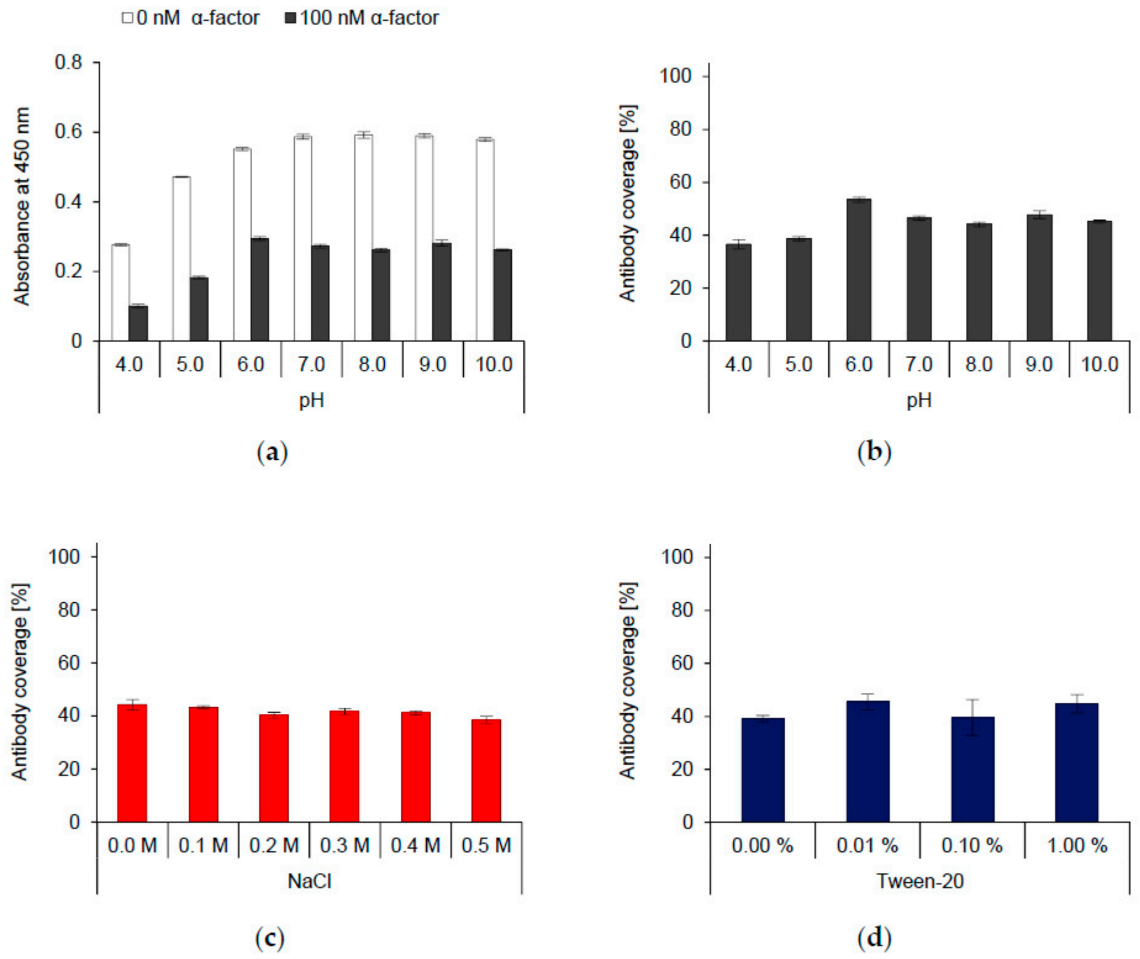

Figure S3. The inverse ELISA under varying conditions. (**a**) Influence of the pH value of the sample on the absorbance values. All buffers contained 0.1 M sodium chloride and 0.01% (*v*/*v*) Tween-20; (**b**) Normalized data regarding the influence of the sample pH. For data normalization, absorbance values corresponding to samples without the pheromone were defined as 100% antibody coverage and absorbance values corresponding to samples containing 100 nM of the α-factor are depicted; (**c**) Influence of the ionic strength on the performance of the inverse ELISA. Sodium chloride at different concentrations was added to phosphate buffers (pH 7.0) containing 0.01% (*v*/*v*) Tween-20. Data were normalized according to (**b**); (**d**) Influence of the detergent concentration on the performance of the inverse ELISA. Tween-20 at different concentrations was added to phosphate buffer (pH 7.0) containing 0.1 M sodium chloride. Data were normalized according to (**b**). All plots correspond to triplicate measurements of at least two independent experiments. Error bars indicate standard deviation.

The inverse ELISA proved to be remarkably robust against changes in the sample matrix composition, similar to the competitive ELISA (Figure S1). Although severely reduced absorbance values were recorded when the initial binding step was performed in acidic conditions, the resulting antibody coverage (obtained upon data normalization, see Materials and Methods) was barely affected (Figure S3a,b). The low absorbance values in acidic environments are most likely caused by electrostatic repulsive forces between the antibodies (positively charged in acidic conditions) and the hydrophobin layer exposing the positively charged $(His)_6$-tag. In contrast, modifying the ionic strength or the detergent concentration during the initial binding step did not have any impact on the sensitivity of the inverse ELISA (Figure S3c,d).

S4. Structure of Authentic and Chimeric Pheromone Genes

Synthesis and secretion of the *S. cerevisiae* α-factor in the fission yeast *S. pombe* was achieved by heterologous expression of an authentic and a chimeric gene [12]. The ORF of *MFα1*, encoding the authentic pheromone precursor of *S. cerevisiae* [13], was heterologously expressed in *S. pombe*. The MFα1p precursor carries four repeats of the mature α-factor sequence (blue) spaced by short stretches of amino acids that contain the processing sites (Figure S4). Additionally, we designed a chimeric gene based on *map2* (encoding the P-factor precursor gene of *S. pombe*). Similar to the *MFα1* gene in *S. cerevisiae*, the *S. pombe map2* gene encodes four repeats of the mature P-factor (red) as part of a precursor protein [13]. Both precursor proteins carry an N-terminal signal peptide (black) for import into the endoplasmic reticulum. Two of the four P-factor repeats of *map2* were replaced by the coding sequence of the *S. cerevisiae* α-factor to obtain the chimeric *map2/MFα1* gene (ENA Accession No. LN554940). Both the authentic *MFα1* and the chimeric ORF were expressed in *S. pombe* under transcriptional control of the *nmt1* promoter.

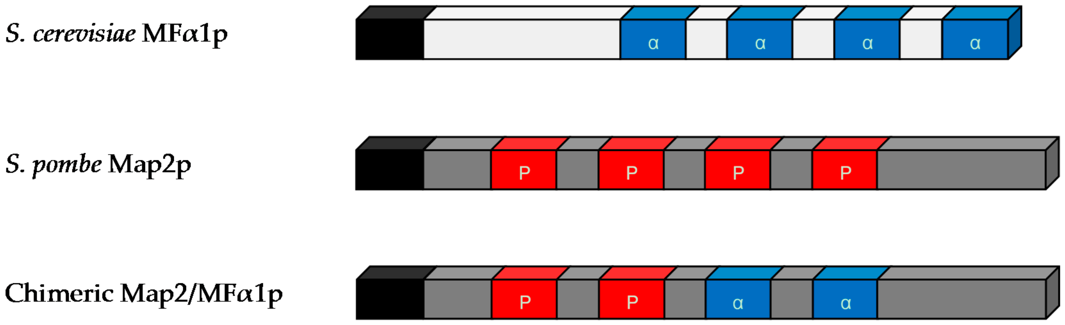

Figure S4. Schematic illustration of authentic and chimeric pheromone precursor proteins. Signal peptides, α-factor copies and P-factor copies are indicated in black, blue and red, respectively.

S5. Selectivity of the ELISA-Based Pheromone Quantification Assay

In order to analyze the selectivity of the ELISA-based approaches for the quantification of the α-factor, we measured the pheromone secretion of *S. cerevisiae* mutant strains lacking either the *KEX1* or the *STE13* gene. These genes encode peptidases involved in the processing of the pheromone precursor and are thus required to ensure pheromone maturation. While Kex1p cleaves off the C-terminal lysine-arginine motif of the α-factor [14], Ste13p is responsible for N-terminal pheromone maturation by removing dipeptide motifs [15]. We therefore assayed the pheromone secretion of these deletion mutants of the mating type α (EUROSCARF, Frankfurt, Germany) by taking samples of respective cultures and analyzing the pheromone concentration in the culture supernatants using the inverse ELISA technique (Figure S5a).

The *ste13* mutant accumulated approximately 10–20 nM of the pheromone within 10–12 h as compared to 30–50 nM of the pheromone secreted by wild type strains in the same time frame. This may hint at a reduced affinity of the α-factor antibody to the N-terminally immature pheromone. Alternatively, the reduced pheromone concentrations compared to the wild type strain may be caused by differences in the growth rate of both strains, as *ste13* mutants were found to grow significantly slower than wild-type cells in minimal medium (data not shown). When pheromone secretion of the *kex1* mutant was analyzed, maximum pheromone concentrations of 5 nM after 10–12 h of incubation were determined (Figure S5a), but only minor differences in the growth rate between wild type and the *kex1* deletion strain were observed. In this deletion strain, C-terminal maturation of the α-factor is not performed. Therefore, the lysine-arginine dipeptide is present at three of the four α-factor repeats encoded by *MFα1*, whereas the fourth pheromone repeat, located at the C-terminal end of the precursor protein, does not carry any C-terminal extension (Figure S4). It is tempting to speculate that the C-terminal lysine-arginine extension interferes with the binding of the antibody, and consequently C-terminally immature pheromones are not measured at all. The

pheromone secretion measured by the inverse ELISA approach might thus result from the fourth pheromone repeat of the MFα1p precursor.

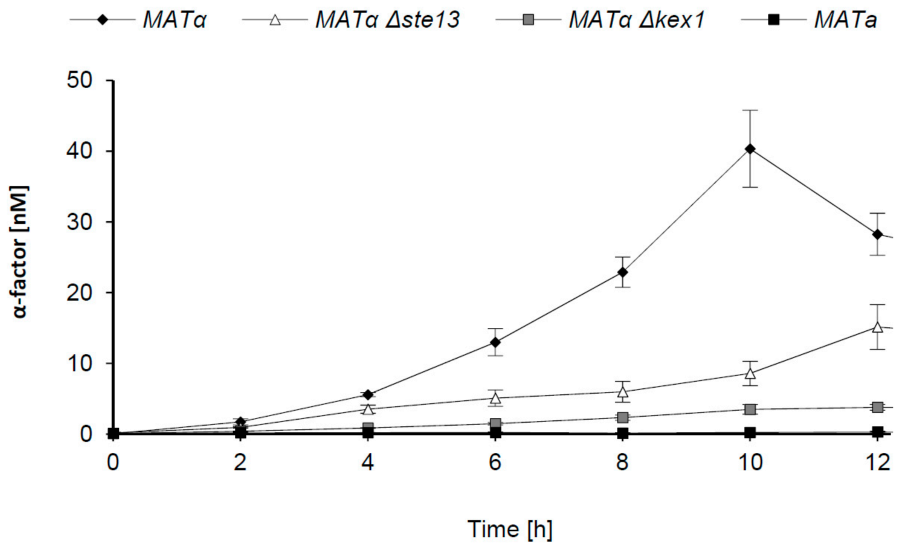

(**a**)

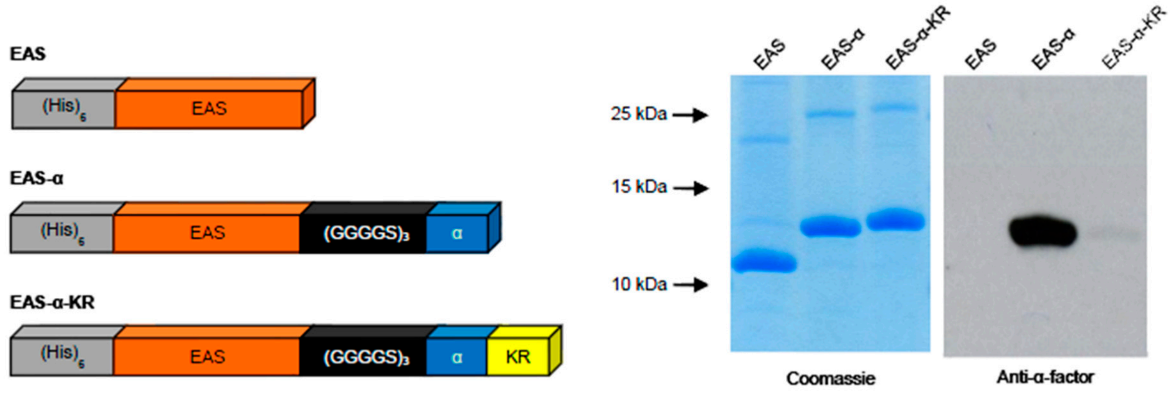

(**b**)

Figure S5. (**a**) Pheromone secretion of wild type and mutant strains of *S. cerevisiae*. Samples of the culture supernatants were taken at the indicated time point and pheromone concentrations were determined using the inverse ELISA approach; (**b**) Schematic illustration of recombinant hydrophobins and separation of purified proteins. The hydrophobins EAS, EAS-α and EAS-α-KR were expressed and purified as detailed in Materials and Methods. A sample of each purified protein fraction was separated by Tricine-SDS-PAGE and visualized by colloidal Coomassie staining or transferred to a PVDF membrane and probed with the α-factor antibody. Molecular weight is indicated at the left.

To further confirm this hypothesis, we cloned, expressed and purified a third hydrophobin variant termed EAS-α-KR (Figure S5b). This protein is based on EAS-α but, in addition, carries a C-terminal lysine-arginine motif, thus resembling the C-terminally immature α-factor secreted by *kex1* mutant strains. When the purified hydrophobin was separated by Tricine-SDS-PAGE and probed with the α-factor antibody, strong differences in the affinity of the antibody to EAS-α and EAS-α-KR were observed (Figure S5b). Taken together, these data strongly suggest that C-terminal processing of the pheromone is mandatory for its measurability by the ELISA-based assays reported here, whereas N-terminally immature pheromone molecules, as secreted by *ste13* deletion strains, can be detected using this approach.

Using these data, a model can be proposed to explain the pheromone secretion of *S. pombe* strains engineered to express *S. cerevisiae MFα1*. There is an apparent discrepancy between high pheromone levels (obtained by inverse ELISA) and a rather low pheromone activity (obtained by pheromone-responding yeast strains [12]) in the supernatants of these cultures. We can exclude that *S. pombe* does not correctly process the C-terminus of the pheromone, as the C-terminally immature pheromone has essentially no pheromone activity [14]. Moreover, as the antibody′s affinity for the C-terminally immature pheromone is severely reduced (see above), the ELISA-based assay largely fails to detect C-terminally immature pheromones. Instead, incomplete N-terminal processing of the α-factor precursor upon expression of *MFα1* in *S. pombe* may account for the observation. In *S. cerevisiae,* the N-terminal processing of the α-factor is carried out by the Ste13p protease [15]. No Ste13p homologue is encoded in the genome of *S. pombe* [16]. The pheromone activity of the N-terminally immature α-factor is at least two orders of magnitude lower than that of the mature form [15], but the N-terminally immature pheromone is detected by the ELISA-based approach (see above). Incomplete N-terminal processing may thus explain the apparent discrepancy between pheromone concentration and activity in supernatants of *MFα1* expressing *S. pombe* strains.

References

1. Wang, Z.; Huang, Y.; Li, S.; Xu, H.; Linder, M.B.; Qiao, M. Hydrophilic modification of polystyrene with hydrophobin for time-resolved immunofluorometric assay. *Biosens. Bioelectron.* **2010**, *26*, 1074–1079.
2. Zhang, M.; Wang, Z.; Wang, Z.; Feng, S.; Xu, H.; Zhao, Q.; Wang, S.; Fang, J.; Qiao, M.; Kong, D. Immobilization of anti-CD31 antibody on electrospun poly(ε-caprolactone) scaffolds through hydrophobins for specific adhesion of endothelial cells. *Coll. Surf. B Biointerfaces* **2011**, *85*, 32–39.
3. Wang, Z.; Lienemann, M.; Qiau, M.; Linder, M.B. Mechanisms of protein adhesion on surface films of hydrophobin. *Langmuir* **2010**, *26*, 8491–8496.
4. Ren, Q.; Kwan, A.H.; Sunde, M. Two forms and two faces, multiple states and multiple uses: Properties and applications of the self-assembling fungal hydrophobins. *Biopolymers* **2013**, *100*, 601–612.
5. Sunde, M.; Kwan, A.H.; Templeton, M.D.; Beever, R.E.; Mackay, J.P. Structural analysis of hydrophobins. *Micron* **2008**, *39*, 773–784.
6. Zampieri, F.; Wösten, H.A.B.; Scholtmeijer, K. Creating surface properties using a palette of hydrophobins. *Materials* **2010**, *3*, 4607–4625.
7. Janssen, M.I.; van Leeuwen, M.B.; van Kooten, T.G.; de Vries, J., Dijkhuizen, L.; Wösten, H.A.B. Promotion of fibroblast activity by coating with hydrophobins in the beta-sheet end state. *Biomaterials* **2004**, *25*, 2731–2739.
8. De Stefano, L.; Rea, I.; Armenante, A.; Giardina, P.; Giocondo, M.; Rendina, I. Self-assembled biofilm of hydrophobins protects the silicon surface in the KOH wet etch process. *Langmuir* **2007**, *23*, 7920–7922.
9. De Vocht, M.L.; Scholtmeijer, K.; van der Vegte, E.W.; de Vries, O.M.; Sonveaux, N.; Wösten, H.A.B.; Ruysschaert, J.M.; Hadziloannou, G.; Wessels, J.G.; Robillard, G.T. Structural characterization of the hydrophobin SC3, as a monomer and after self-assembly at hydrophobic/hydrophilic interfaces. *Biophys. J.* **1998**, *74*, 2059–2068.
10. Gunning, A.P.; De Groot, P.W.J.; Visserb, J.; Morris, V.J. Atomic force microscopy of a hydrophobin protein from the edible mushroom *Agaricus Bisporus*. *J. Coll. Interf. Sci.* **1998**, *201*, 118–126.
11. Wösten, H.A.B.; Schuren, F.H.J.; Wessels, J.G.H. Interfacial self-assembly of a hydrophobin into an amphipathic protein membrane mediates fungal attachment to hydrophobic surfaces. *EMBO J.* **1994**, *13*, 5848–5854.
12. Hennig, S.; Clemens, A.; Rödel, G.; Ostermann, K. A yeast pheromone-based inter-species communication system. *Appl. Microbiol. Biotechnol.* **2015**, *99*, 1299–1308, doi:10.1007/s00253-014-6133-5.
13. Davey, J.; Davis, K.; Hughes, M.; Ladds, G.; Powner, D. The processing of yeast pheromones. *Semin Cell Dev Biol* **1998**, *9*, 19-30, doi:10.1006/scdb.1997.0197.
14. Dmochowska, A.; Dignard, D.; Henning, D.; Thomas, D.Y.; Bussey, H. Yeast *KEX1* gene encodes a putative protease with a carboxypeptidase B-like function involved in killer toxin and alpha-factor precursor processing. *Cell* **1987**, doi:10.1016/0092-8674(87)90030-4.

15. Julius, D.; Blair, L.; Brake, A.; Sprague, G.; Thorner, J. Yeast alpha factor is processed from a larger precursor polypeptide: The essential role of a membrane-bound dipeptidyl aminopeptidase. *Cell* **1983**, doi:10.1016/0092-8674(83)90070-3.
16. Wood, V.; Gwilliam, R.; Rajandream, M.A.; Lyne, M.; Lyne, R.; Stewart, A.; Sgouros, J.; Peat, N.; Hayles, J.; Baker, S.; *et al*. The genome sequence of *Schizosaccharomyces pombe*. *Nature* **2002**, doi:10.1038/nature724.
